# Wave Propagation in a Fractional Viscoelastic Tissue Model: Application to Transluminal Procedures

**DOI:** 10.3390/s21082778

**Published:** 2021-04-15

**Authors:** Antonio Gomez, Guillermo Rus, Nader Saffari

**Affiliations:** 1 UCL Mechanical Engineering, University College London, London WC1E 7JE, UK; n.saffari@ucl.ac.uk; 2 Instituto de Investigación Biosanitaria, ibs.GRANADA, 18012 Granada, Spain; 3 Structural Mechanics Department, University of Granada, 18071 Granada, Spain; grus@ugr.es; 4 Excellence Research Unit “ModelingNature” (MNat), University of Granada, 18071 Granada, Spain

**Keywords:** transluminal elastography, shear wave, fractional viscoelasticity, kelvin voigt fractional derivative, finite difference

## Abstract

In this article, a wave propagation model is presented as the first step in the development of a new type of transluminal procedure for performing elastography. Elastography is a medical imaging modality for mapping the elastic properties of soft tissue. The wave propagation model is based on a Kelvin Voigt Fractional Derivative (KVFD) viscoelastic wave equation, and is numerically solved using a Finite Difference Time Domain (FDTD) method. Fractional rheological models, such as the KVFD, are particularly well suited to model the viscoelastic response of soft tissue in elastography. The transluminal procedure is based on the transmission and detection of shear waves through the luminal wall. Shear waves travelling through the tissue are perturbed after encountering areas of altered elasticity. These perturbations carry information of medical interest that can be extracted by solving the inverse problem. Scattering from prostate tumours is used as an example application to test the model. In silico results demonstrate that shear waves are satisfactorily transmitted through the luminal wall and that echoes, coming from reflected energy at the edges of an area of altered elasticity, which are feasibly detectable by using the transluminal approach. The model here presented provides a useful tool to establish the feasibility of transluminal procedures based on wave propagation and its interaction with the mechanical properties of the tissue outside the lumen.

## 1. Introduction

Palpation has been used since ancient times as a technique for evaluating hardness due to abnormal tissue [1]. It is broadly known that some pathologies manifest as an alteration in the elastic properties of the affected tissues, for example liver fibrosis, steatosis, and many types of cancerous tumours. Elastography is a family of imaging modalities that evaluates the elasticity of tissue for medical diagnosis. First elastography techniques were developed in the late 1980s to early 1990s with the aim of improving ultrasound-based imaging methods [2,3,4,5]. Conventional ultrasound differentiates body structures based on the changes of the acoustic impedance, which in turn depends on the bulk modulus of the tissue. However, the variation of the bulk modulus for soft tissue is significantly less than an order of magnitude [6,7,8]. On the other hand, elastography senses the deformation of tissue that ultimately depends on the value of the shear modulus, which varies over several order of magnitude for soft tissue [8,9].

Many clinical applications of elastography are performed from outer surfaces of the body using surface ultrasound probes, for instance, in liver fibrosis assessment and breast cancer detection. However, there are cases where the target is better accessible from a body lumen. Some of the most relevant examples of this are intravascular elastography and transrectal elastography of the prostate. Intravascular elastography using Strain Elastography (SE) (also known as Intravascular Ultrasonic Palpation) [10,11], Acoustic Radiation Force (ARF) imaging [12,13], and more recently Shear Wave Elastography (SWE) [14,15], have been widely investigated with the aim of assessing the rupture risk of lipid plaques in atherosclerosis. Transrectal elastography of prostate for cancer detection is also a highly active front with numerous published studies using different elastography approaches [16]. Amongst all these, the most widely used is SWE, which has been found to improve the efficiency of prostate cancer diagnosis [17,18]. Other examples of applications of transluminal elastography can be found in transvaginal, endoscopic and gastrointestinal imaging [19].

Modelling the mechanical response of soft tissue is often required in elastography. Soft tissue is well known for behaving viscoelastically. Classical linear viscoelastic constitutive models, such as Zener, Kelvin Voigt (KV) and Maxwell, have been extensively used, however, theses models are based on single relaxation processes, which are not representative of the soft tissue mechanical response [20,21]. Fractional linear viscoelastic models, such as the Fractional Zener, the KVFD or the spring-pot, overcome this limitation by reproducing the power law behaviour of cumulative multiple relaxation processes [21].

On the other hand, it is well known that absorption as a function of frequency, for both compressional and shear waves, often follows the power law [22]:

(1) $$\alpha_{k}\left(\omega \right)=\alpha_{0}\omega^{y}$$

where $\alpha_{k}\left(\omega \right)$ is the absorption law as a function of frequency, $\alpha_{0}$ is the absorption coefficient at a linear frequency $f=1/\left(2\pi \right)$, ω is the angular frequency, and *y* is the power law exponent. The exponent *y* of the power law (Equation (1)) takes values from 0 to 2 depending on the frequency region of the application compared with the relaxation time τ. The low-frequency region, where ωτ≪1, usually can be found in ultrasound imaging, with exponent *y* between 1 and 2 [23]. On the other hand, shear waves in elastography applications fall within both the low- and high-frequency region, experiencing absorption laws with exponent *y* from less than 1 to 2 [22]. The classical viscoelastic models can only model limited values of exponents for power law absorption [24]. In the case of the KV-based wave equation models absorption with *y* = 2 for the low-frequency region, and *y* = 0.5 for the high-frequency region. Fractional order wave equations have been proved to fill these gaps and produce models that fit well experimental absorption power laws with a variable exponent [25]. Many studies have suggested the use of a fractional generalised version of the KV law for modelling elastography [22,23,26,27], the KVFD constitutive law. Furthermore, Holm and Näsholm [23] demonstrated that some of the most used fractional wave equations, such as the fractional Szabo equation and the fractional Laplacian wave equation, are low-frequency approximations of the KVFD wave equation. According to the KVFD law, the stress σ depends on the fractional time derivative of order α of the strain ϵ, as shown in the following equation for the shear stress case:

(2) $$\sigma =\mu \epsilon +\eta_{s}\frac{\partial^{\alpha }\epsilon }{\partial t^{\alpha }}$$

where μ is the second Lamé’s parameter and $\eta_{s}$ is the shear viscosity. μ is also known as the shear modulus.

Some examples of wave propagation modelling using KVFD are the studies of Caputo et al. [26], Zhang and Holm [27], and Sinkus et al. [28]. Caputo et al. [26] modelled compressional waves for biological applications, assuming a KVFD constitutive law. The model was *in silico* validated against an analytical solution in a homogeneous breast fatty tissue-like medium. Using a similar approach, Zhang and Holm [27] modelled in 1D shear waves generated by ARF in different viscoelastic media.

In this paper, a forward model is presented as the first step in the development of a new transluminal procedure for performing elastography. The examination of the propagation mechanism outside the lumen is key for further investigating the feasibility of the procedure. The model is based on a Kelvin Voigt Fractional Derivative (KVFD) wave equation solved by a Finite Difference Time Domain (FDTD) scheme. An *in silico* example based on prostate cancer detection is built to test the wave propagation model and illustrate the new transluminal approach. The novelty of this work is therefore the application of the KVFD forward model to the new transluminal procedure.

## 2. Materials and Methods

### 2.1. A New Transluminal Elastography Approach

**Figure 1:**
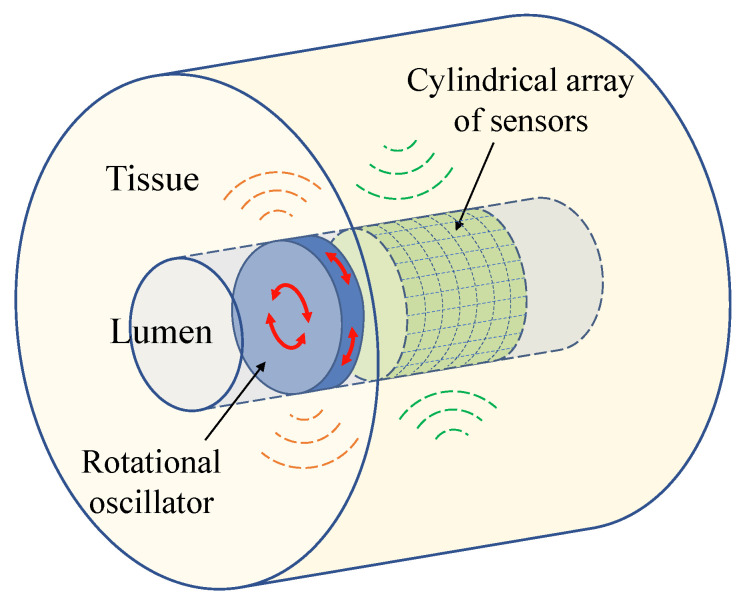
Conceptual idealisation of the transluminal elastography approach. The transluminal probe, composed for at least one rotational oscillator and a cylindrical array of sensors, is inserted through the lumen.

**Figure 2:**
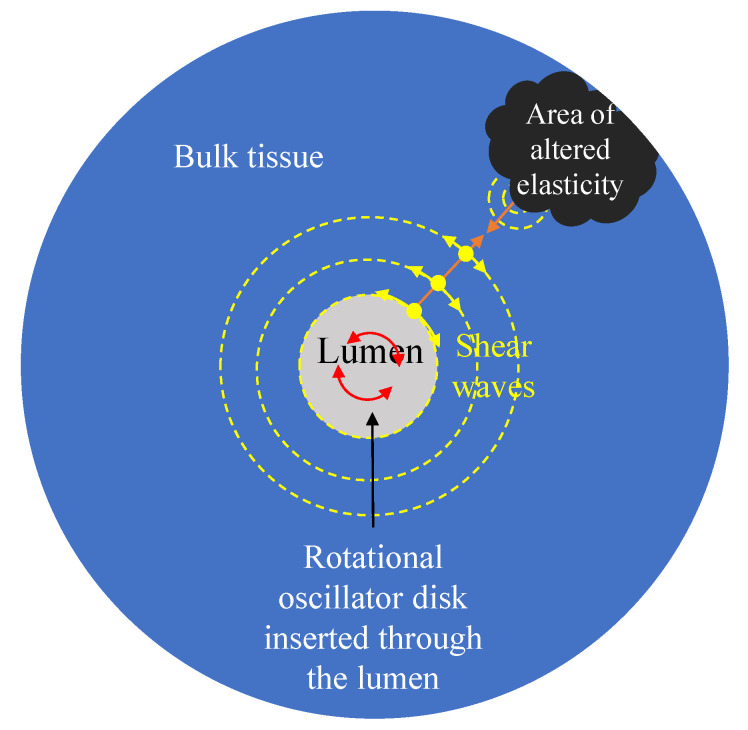
Cross section scheme of the proposed transluminal elastography approach. Shear waves propagates radially (orange arrow) from the rotational oscillator disk. Particles vibrate in an arc-shaped manner perpendicular to the propagation (yellow arrows). Echoes are generated as the shear waves interact with the area of altered elasticity.

The new transluminal procedure is based on the transmission of shear waves and the detection of its echoes through the luminal wall [29]. Figure 1 and Figure 2 show the conceptual idealisation of the approach. The rotational oscillation of a disk in contact with the luminal wall creates a pseudo-spherical pattern of shear waves that interacts with the tissue architecture. Echoes are created when the wavefront encounters an area of altered elasticity. This method of transmission minimises the generation of undesired compressional waves and yields a particular arc-shape particle vibration pattern (see Figure 2). The rotational oscillatory displacement is applied along the whole circumference of the contact surface between the disk and lumen wall. This induces shear stress acting tangentially to the urethral wall along its whole circumferential dimension, while avoiding compressional stress in any direction. Three previous studies did not observe the presence of any compressional waves using a similar wave generation mechanism [30,31,32].

Some general inherent advantages of this new transluminal approach over other elastography techniques may be: (1) The ability of reaching deep organs by using body cavities, thus getting closer and eliminating possible obstacles in the way to the target and (2) the possibility of using higher frequencies, e.g., above 500 Hz, thus improving the image resolution and the capacity of detecting smaller targets, although there is a trade-off against a higher attenuation and potentially a poorer Signal-to-Noise Ratio (SNR). Although the frequency of shear waves generated by most commercially available SWE systems is in the range between 50 and 400 Hz, the generation of shear waves at higher frequencies is feasible [33]. For example, as observed in ex vivo porcine liver, frequencies up to 1 kHz can be excited using Shear Wave Dispersion Ultrasound Vibrometry (SDUV) [34,35]. The practical frequency range of the transluminal procedure will nevertheless be determined by the attenuation of the medium and the sensitivity of the probe; (3) the possibility of simultaneous 3D scanning; (4) flexibility of the probe design, thus producing low deformation of tissue; and (5) low levels of energy, and therefore a lower thermal index compared with ARF-based techniques. Other particular advantages could also be found depending on the clinical application.

### 2.2. Wave Propagation Model

#### 2.2.1. Model Geometry and Equations

The equations that govern the propagation of mechanical waves can be described by the classical Navier equation in an isotropic elastic solid medium [36], in vector notation:

(3) $$\rho \frac{\partial^{2}u}{\partial t^{2}}=\left(\lambda +\mu \right)\nabla \left(\nabla \cdot u\right)+\mu \nabla^{2}u+f$$

where ρ is the local density of the medium, u is the vector of displacements, λ is the first Lamé’s parameter, and f is the external body force vector.

Equation (3) can be obtained by combining three different types of equations: The linear conservation of momentum (Equation (4)), the strain-displacement linear relationship (Equation (5)), and a constitutive law (also known as the material law or rheological law) (Equation (6)) [36]. Index notation is hereinafter used:

(4) $$\frac{\partial^{2}u_{i}}{\partial t^{2}}=\frac{\partial^{2}\sigma_{ij}}{\partial x_{j}}+f_{i}$$

(5) $$\epsilon_{ij}=\frac{1}{2}\left(\frac{\partial u_{j}}{\partial x_{i}}+\frac{\partial u_{i}}{\partial x_{j}}\right)$$

(6) $$\sigma_{ij}=\lambda \delta_{ij}\epsilon_{kk}+2\mu \epsilon_{ij}$$

where i,j are the index components, *u* is the displacement field, σ is the stress tensor, *x* represents the spatial variables, *f* is the external body force, ϵ is the strain tensor, and δ is the Kronecker delta.

**Figure 3:**
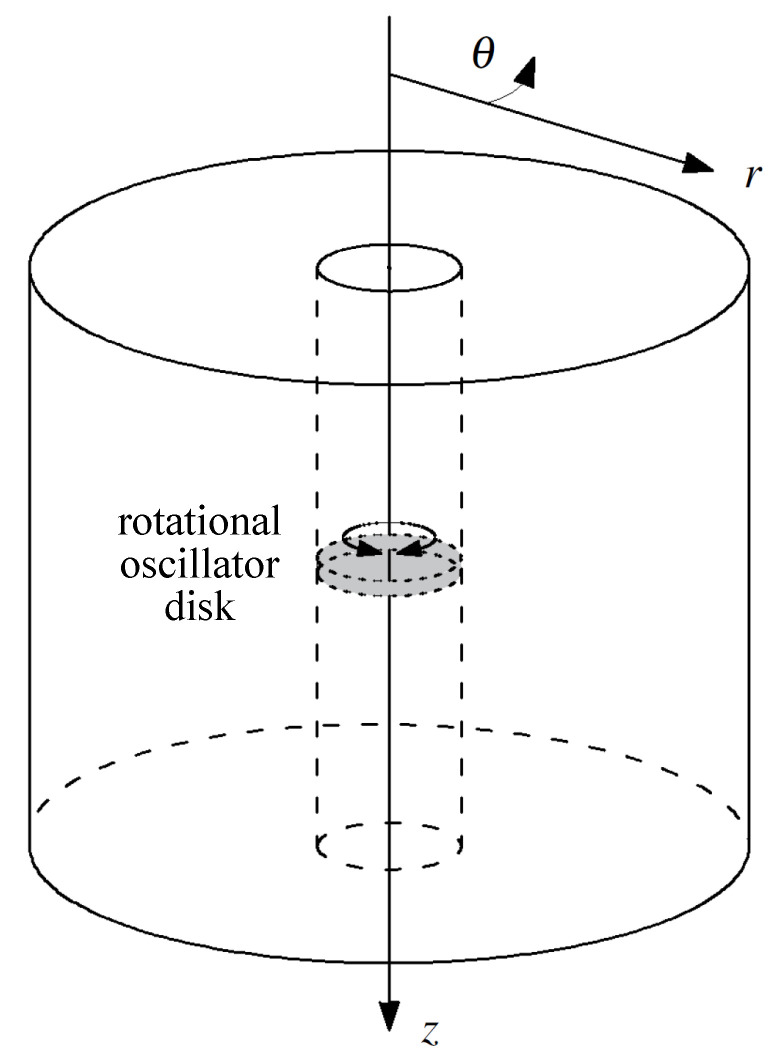
Geometry and system of coordinates used in the wave propagation model. The grey disk represents an emitter.

The spatial domain of the model consisted in a solid cylinder containing a coaxial straight lumen-like conduit (Figure 3). A cylindrical coordinate system (r,θ,z) was considered as indicated in Figure 3.

The conservation of momentum equations in cylindrical coordinates are as follows [37]:

(7) $$\rho \frac{\partial^{2}u_{r}}{\partial t^{2}}=\frac{\partial \sigma_{rr}}{\partial r}+\frac{1}{r}\frac{\partial \sigma_{r\theta }}{\partial \theta }+\frac{\partial \sigma_{rz}}{\partial z}+\frac{1}{r}\left(\sigma_{rr}-\sigma_{\theta \theta }\right)+f_{r}$$

(8) $$\rho \frac{\partial^{2}u_{\theta }}{\partial t^{2}}=\frac{\partial \sigma_{r\theta }}{\partial r}+\frac{1}{r}\frac{\partial \sigma_{\theta \theta }}{\partial \theta }+\frac{\partial \sigma_{\theta z}}{\partial z}+\frac{2}{r}\sigma_{r\theta }+f_{\theta }$$

(9) $$\rho \frac{\partial^{2}u_{z}}{\partial t^{2}}=\frac{\partial \sigma_{rz}}{\partial r}+\frac{1}{r}\frac{\partial \sigma_{\theta z}}{\partial \theta }+\frac{\partial \sigma_{zz}}{\partial z}+\frac{1}{r}\sigma_{rz}+f_{z}$$

where *u* is the displacement of particles and *f* the external forces. Suffixes r,θ,z represents the three components of each magnitude according the system of coordinates.

The linear strain-displacement relationships are as follows [36]:

(10) $$\epsilon_{rr}=\frac{\partial u_{r}}{\partial r}$$

(11) $$\epsilon_{\theta \theta }=\frac{1}{r}\frac{\partial u_{\theta }}{\partial \theta }+\frac{u_{r}}{r}$$

(12) $$\epsilon_{zz}=\frac{\partial u_{z}}{\partial z}$$

(13) $$\epsilon_{r\theta }=\frac{1}{2}\left(\frac{1}{r}\frac{\partial u_{r}}{\partial \theta }+\frac{\partial u_{\theta }}{\partial r}-\frac{u_{\theta }}{r}\right)$$

(14) $$\epsilon_{rz}=\frac{1}{2}\left(\frac{\partial u_{z}}{\partial r}+\frac{\partial u_{r}}{\partial z}\right)$$

(15) $$\epsilon_{\theta z}=\frac{1}{2}\left(\frac{\partial u_{\theta }}{\partial z}+\frac{1}{r}\frac{\partial u_{z}}{\partial \theta }\right).$$

Viscoelasticy implies that the mechanical response of tissue also depends on the viscosity. Therefore, the elastic constitutive law (Equation (6)) is not suitable and a viscoelastic law, the KVFD law in this case, is used. The equations for a KVFD constitutive law [26] in cylindrical coordinates are:

(16) $$\sigma_{rr}=\left(\lambda +\eta_{p}\frac{\partial^{\alpha_{p}}}{\partial t^{\alpha_{p}}}\right)\left(\epsilon_{rr}+\epsilon_{\theta \theta }+\epsilon_{zz}\right)+2\left(\mu +\eta_{s}\frac{\partial^{\alpha }}{\partial t^{\alpha }}\right)\epsilon_{rr}$$

(17) $$\sigma_{\theta \theta }=\left(\lambda +\eta_{p}\frac{\partial^{\alpha_{p}}}{\partial t^{\alpha_{p}}}\right)\left(\epsilon_{rr}+\epsilon_{\theta \theta }+\epsilon_{zz}\right)+2\left(\mu +\eta_{s}\frac{\partial^{\alpha }}{\partial t^{\alpha }}\right)\epsilon_{\theta \theta }$$

(18) $$\sigma_{zz}=\left(\lambda +\eta_{p}\frac{\partial^{\alpha_{p}}}{\partial t^{\alpha_{p}}}\right)\left(\epsilon_{rr}+\epsilon_{\theta \theta }+\epsilon_{zz}\right)+2\left(\mu +\eta_{s}\frac{\partial^{\alpha }}{\partial t^{\alpha }}\right)\epsilon_{zz}$$

(19) $$\sigma_{r\theta }=2\left(\mu +\eta_{s}\frac{\partial^{\alpha }}{\partial t^{\alpha }}\right)\epsilon_{r\theta }$$

(20) $$\sigma_{rz}=2\left(\mu +\eta_{s}\frac{\partial^{\alpha }}{\partial t^{\alpha }}\right)\epsilon_{rz}$$

(21) $$\sigma_{\theta z}=2\left(\mu +\eta_{s}\frac{\partial^{\alpha }}{\partial t^{\alpha }}\right)\epsilon_{\theta z}$$

where, $\eta_{p}$ is the bulk KVFD viscous parameter and $\alpha_{p}$ is the order of the fractional derivative for the volumetric components of the strain. The range of values of α goes from 0 to 2, but the most common values in dynamic elastography are lower than 1 [27].

Axial symmetry was taken into account to reduce the spatial domain of the model. The wavefront generated by the rotational oscillator propagates axisymmetrically (see Figure 2). This fact, together with the axisymmetric geometry of the model, yielded a reduction in the displacement field to only one component, the angular displacement $u_{\theta }$. The resulting 2D domain after the simplification is an r-z plane. The system of equations of conservation of momentum was reduced to Equation (22), the strain-displacement relationships were reduced to Equations (23) and (24), and the KVFD constitutive law was reduced to Equations (19) and (21).

(22) $$\rho \frac{\partial^{2}u_{\theta }}{\partial t^{2}}=\frac{\partial \sigma_{r\theta }}{\partial r}+\frac{\partial \sigma_{\theta z}}{\partial z}+\frac{2}{r}\sigma_{r\theta }$$

(23) $$\epsilon_{r\theta }=\frac{1}{2}\left(\frac{\partial u_{\theta }}{\partial r}-\frac{u_{\theta }}{r}\right)$$

(24) $$\epsilon_{\theta z}=\frac{1}{2}\frac{\partial u_{\theta }}{\partial z}.$$

The boundary conditions of the problem were: The excitation source at the points on the luminal wall where the rotational oscillator disk is placed (Equation (25)), and the absence of shear stress on the rest of the luminal wall as a free boundary (Equations (26) and (27)).

(25) $$u_{\theta }\left(r_{lumen},z_{emitter}\right)=u_{excitation}$$

(26) $$\sigma_{rz}\left(r_{lumen},z\notin z_{emitter}\right)=0$$

(27) $$\sigma_{\theta z}\left(r_{lumen},z\notin z_{emitter}\right)=0.$$

**Figure 4:**
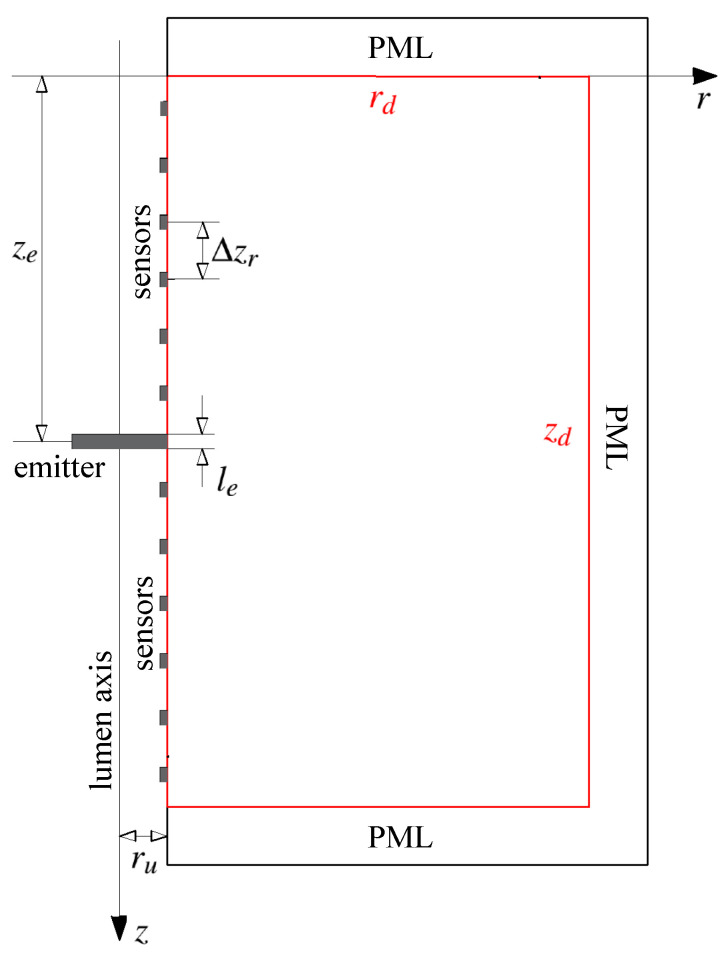
Scheme of the geometrical parameters of the wave propagation model. Real spatial domain contoured in red. Perfectly Matched Layer (PML) boundary conditions for absorbing undesired reflections were set at the edges with the exception of the luminal wall. Rotational oscillator disk in grey in contact with the luminal wall.

All the relevant geometrical elements of the model are shown in Figure 4. The description and set value of these parameters are detailed in Section 3. Depending on each specific situation, absorption boundaries can be set at the remaining edges of the domain in order to fade out undesired reflected waves, for instance, by using Perfectly Matched Layer (PML).

#### 2.2.2. Numerical Method

A FDTD approach was chosen for modelling the propagation of shear waves. An elastic model based on FDTD was developed by Gomez et al. [38] for a transurethral elastography approach. The model used a velocity-stress formulation adapted from Virieux [39]. This approach reduced the amount of time derivatives, and therefore the computational overhead. However, the history of the displacement field needs to be stored for computing the fractional derivative as demonstrated further below. For this reason, a displacement-stress formulation, composed by Equations (19) and (21)–(24), is chosen. Strain-displacement relationship equations (Equations (23) and (24)) are fused into the KVFD constitutive equations (Equations (19) and (21)), thus reducing the memory required for computing the algorithm.

**Figure 5:**
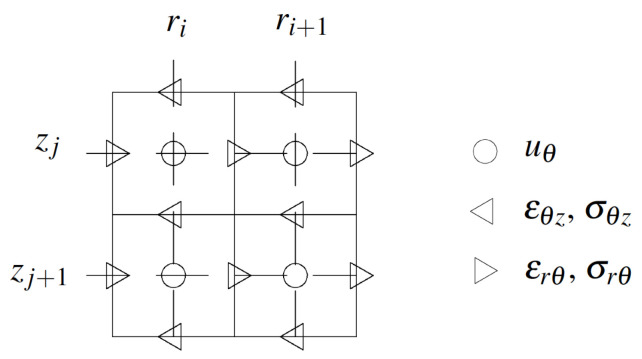
Staggered grid discretisation showing the locations of variables: Displacements ($u_{\theta }$), stresses ($\sigma_{r\theta }$, $\sigma_{\theta z}$) and strains ($\epsilon_{r\theta }$, $\epsilon_{\theta z}$).

Spatial discretisation was achieved by a staggered grid as illustrated in Figure 5 [38]. The displacement component was placed at grid positions that are offset by a half-step from the corresponding stress and strain components. Space was uniformly sampled, with r=iΔr/2 and z=jΔz/2 for integers i,j and space step of discretisation Δr and Δz.

Time was uniformly sampled via t=nΔt for an integer *n* and a time step Δt. All stress, strain, and displacement components were computed at the same time value, thus providing verifiable magnitudes at each time step. Medium properties, such as density and KVFD viscoelastic parameters, were introduced into the model by setting their values at the grid cells of the discretised space domain.

In order to apply the FDTD method to the system of equations (Equations (19) and (21)–(24)), the expressions (28)–(31) were used. These expressions were derived from Taylor series expansions and details can be found in general FDTD literature. The centred finite difference scheme was chosen for the derivatives with respect to one of the spatial variables. In this case, the staggered grid yielded a second order approximation of the derivative:

(28) $${\left.\frac{\partial g(x,t)}{\partial x}\right|}_{x_{i},t_{n}}=\frac{g\left(x_{i+1/2},t_{n}\right)-g\left(x_{i-1/2},t_{n}\right)}{\Delta x}+O\left(\Delta x^{2}\right)$$

where *g* is an arbitrary differentiable function within the domain of interest, *x* represents one of the spatial variables *r* and *z*, *t* represents time, and Δx is one of the spatial steps used for the spatial discretisation.

For the first and the second order time derivatives, backward finite differences were chosen, since during the computation, the future states of the functions were unknown:

(29) $${\left.\frac{\partial g(x,t)}{\partial t}\right|}_{x_{i},t_{n+1}}=\frac{g\left(x_{i},t_{n+1}\right)-g\left(x_{i},t_{n}\right)}{\Delta t}+O\left(\Delta t\right).$$

(30) $${\left.\frac{\partial^{2}g\left(x,t\right)}{\partial t^{2}}\right|}_{x_{i},t_{n+1}}=\frac{g\left(x_{i},t_{n+1}\right)-2g\left(x_{i},t_{n}\right)+g\left(x_{i},t_{n-1}\right)}{\Delta t^{2}}+O\left(\Delta t^{2}\right)$$

For the fractional derivatives of order α, a backward difference formulation based on the Grünwald–Letnikov (GL) approximation was chosen [40,41]:

(31) ∂αg(x,t)∂tαxi,tn+1=∑k=0N(−1)kΔtααkgxi,tn−k+1+OΔt

where *N* is the maximum value for *n*. The derivation of Equation (31) can be found in Carcione et al. [41].

As can be noticed by analysing Equation (31), the approximated value of the derivative is given in terms of the summation of all previous states of the function g(x,t). This summation of states leads to an iterative and storing process that may result in huge memory consumption. The binomial coefficients that appear in the expression are negligible for *k* exceeding an integer *J*. This allows the application of the so-called short-memory principle, through which the summation can be truncated at k=L, with L≤N being the so-called effective memory length [40].

Higher order approximations for the derivatives could have been used to reduce truncation errors. However, higher order schemes require the computation of more space-time grid nodes. This generates complications around the boundaries, where extra grid nodes have to be added to satisfy the high order approximation scheme. Nevertheless, by using small enough values of the space and time steps, Δx and Δt, the truncation errors are also reduced, as can be deduced from Equations (28)–(31). The discrete equations derived from the application of the FDTD expressions can be found in Appendix A.

Two types of numerical errors may occur when modelling with FDTD methods. The first type is linked to the spatial steps of the discretisation and generates phase errors known as numerical dispersion [42]. Gomez et al. [38] considered a conservative rate of a minimum of 20 space intervals per wavelength λ. However, the FDTD model employed was elastic. In viscoelastic cases, since viscoelasticity naturally shows dispersion effects, an appropriate verification test for numerical dispersion errors must be carried out. The second type is linked to the time step of the temporal discretisation. It affects the stability of the wave amplitude during the simulation. In the case of fractional derivatives, the inference of the criterion is not immediate and requires a thorough mathematical development. As an alternative, a trial-and-error approach was used. Additionally, an appropriate analysis to avoid errors due to a poor implementation of the short-memory principle must also be carried out.

The rotational oscillator disk was not physically modelled. Instead, the excitation displacement signal was directly implemented at the mesh elements of the luminal wall where the disk would be placed. Similarly, the array of sensors was not modelled, instead, the displacement values at the mesh elements in physical contact with the sensors’ locations were recorded.

A PML for absorbing the incoming reflections from the outer boundaries, was incorporated surrounding the spatial domain (see Figure 4). The PML scheme was adapted from the formulation in cylindrical coordinates developed by Liu [43] and was directly merged into the discrete equations of the problem. Details of the PML formulation can be observed in the Appendix A.

The FDTD wave propagation model was implemented in MATLAB^®^ (Release 2017a) using the Parallel Computing Toolbox^™^ (Release 2017a, MathWorks, Natick, United States).

## 3. Results

The transluminal wave propagation model was tested on a example case of potential clinical utility: Imaging of prostate cancer using a transurethral elastography approach.

### 3.1. Prostate Cancer

Worldwide, prostate cancer is the second most common cancer in men and the fifth leading cause of death from cancer, with an estimation of 1.3 million men diagnosed and 360,000 associated deaths worldwide in 2018 [44]. It has been shown that prostatic cancerous nodules are usually stiffer than adjacent normal prostatic tissue, which suggests great potential for elastography to identify prostate cancer [45,46]. Furthermore, correlation between stiffness and the Gleason score, a grading system (from 2 to 10) used to evaluate the prognosis of men with prostate cancer, have been observed. The higher the score, the more aggressive the tumour, and according to some studies, the higher the stiffness [47,48,49]. The clinical use of elastography for imaging the prostate has mainly been carried out by SE and SWE [50,51].

Shear wave scattering from prostate cancer is here used as an example of potential application for the new transluminal procedure. Since shear waves are sensitive to changes in the elastic properties of the tissue, techniques based on these waves are particularly well suited for detecting and characterising regions of elevated stiffness in the prostate [38]. In this particular case, the data for future image reconstruction would be based on the reception at the urethral wall of shear wave echoes generated by stiff areas in the gland that can be associated with cancer. Some overlap may be expected since other benign pathologies, such as Benign Prostatic Hyperplasia (BPH) and acute and chronic inflammation, also show elevated stiffness. Nevertheless, cancerous tumours show the highest stiffness variation compared with normal surrounding tissue [48,52]. The signals detected at the urethral wall carry information about the medium of propagation and the stiff areas located within it, i.e., features of the stiff lesion, such as location, size, and viscoelastic properties.

While the proposed approach uses the urethral passage, current elastography techniques are transrectal. For this reason, one additional potential advantage of the transurethral approach is the possibility of monitoring transrectally delivered therapies that yields changes in the elasticity of the treated tissue. A good example of this is High Intensity Focused Ultrasound (HIFU) ablation of prostate cancer [53].

**Figure 6:**
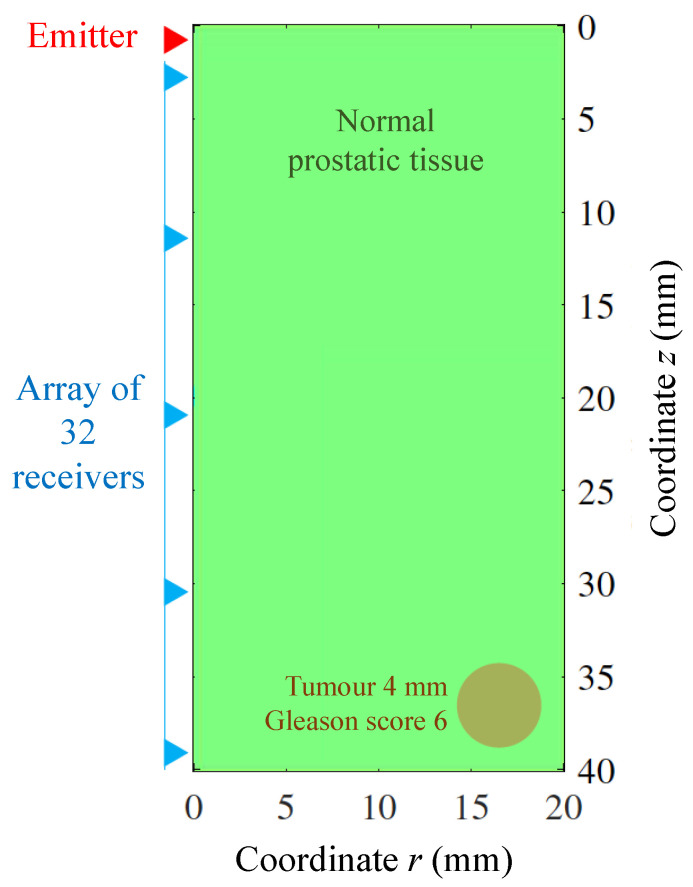
A particular clinical scenario used to test the wave propagation model for transluminal elastography imaging of prostate cancer. The rotational oscillator emitter was set at the top end of the urethral conduit. The remaining urethral wall was used for placing the array of receivers.

A particular clinical scenario was used to test the wave propagation model. The simulated scenario consisted in a normal prostate-like medium that contained a tumour located at the longest possible distance with respect to the emitter (see Figure 6). The size and mechanical properties of the tumour were estimated from elastography studies for a minimum clinically significant prostate cancer. This was a tumour of 4 mm with a Gleason score of 6 [54]. Additionally, the stability analysis of the model, as well as the study for the numerical dispersion and the error due to the short-memory principle, were addressed. The dimensions of the model (see Figure 6 and Table 1) were chosen according to the usual size of human prostates with pathological conditions [55,56]. Although the diameter of the human male urethra is variable along its length (from 4 mm to 10 mm), its value was taken constant at 6.50 mm as in other models from the literature [57,58]. It is clear that any area of altered elasticity in the 2D geometry will be translated to the 3D space as a solid of revolution, in this case a toroid. Although this is not representative of prostate cancer it is considered admissible for the testing purpose of this work.

### 3.2. KVFD Viscoelastic Parameters of Prostatic Tissue

Shear mechanical properties for normal and cancerous prostatic tissue based on the KVFD model (μ, $\eta_{s}$, and α) were required for simulating the clinical scenario. For convenience, the shear KVFD viscous parameter is hereinafter denoted as a simple η. The values of theses parameters were inferred by combining data from several elastography studies [27,47,48,52,59,60,61] as no clear and consistent values were found after a literature review. As can be seen in Equation (32), the KVFD expression for the complex shear modulus $G^{*}$ is a function of the three shear KVFD parameters:

(32) $$G^{*}\left(\omega \right)=\mu +\eta {\left(i\omega \right)}^{\alpha }.$$

The shear modulus of normal tissue, taken as the absolute value of the complex shear modulus $G^{*}$, is expected to take values from 2 to 10 kPa [47,48,52,59].

The parameter α was set as 0.35, which is representative of the findings by Zhang et al. [59] and Zhang and Holm [27]. The rest of shear KVFD parameters were estimated to match the expected range of absolute values of the complex shear modulus. In the studies by Zhang and Holm [27] and Mitri et al. [60], the ratio of the elastic μ and viscous η parameters was found to be of two orders of magnitude. For this reason, and also to produce a velocity dispersion curve resembling that provided by Mitri et al. [60], μ and η were chosen to be 3.0 kPa and 35 Pa·s${}^{\alpha }$, respectively.

The shear phase velocity of a monochromatic plane shear wave according the KVFD model can be derived from the combination of the following equations:

(33) $$\rho c_{s}^{2}\left(\omega \right)=\frac{2\left(G^{\prime 2}\left(\omega \right)+G^{\prime \prime 2}\left(\omega \right)\right)}{G^{\prime }\left(\omega \right)+\sqrt{G^{\prime 2}\left(\omega \right)+G^{\prime \prime 2}\left(\omega \right)}}$$

with [27]:

(34) $$G^{\prime }\left(\omega \right)=\mu +\eta \omega^{\alpha }\cos\left(\frac{\alpha \pi }{2}\right)$$

(35) $$G^{\prime \prime }\left(\omega \right)=\eta \omega^{\alpha }\sin\left(\frac{\alpha \pi }{2}\right)$$

where ρ is the density, and $G^{\prime }$ and $G^{\prime \prime }$ are the real and imaginary parts of the complex shear modulus $G^{*}$, respectively.

**Figure 7:**
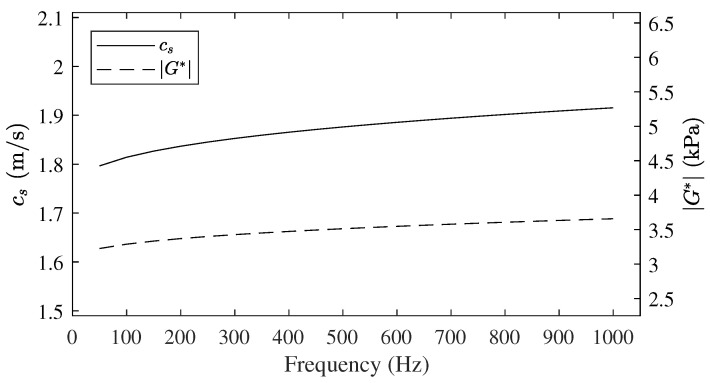
Shear velocity dispersion curve $c_{s}$ and absolute value of the complex shear modulus $G^{*}$, for the shear Kelvin Voigt Fractional Derivative (KVFD) parameters inferred for normal prostatic tissue.

Tissue density, ρ, was considered to be 1000 kg/m^3^. The combination of the three shear KVFD parameters produces values of the shear modulus ranging from 3.2 to 3.6 kPa (according to Equation (32)) and velocities from 1.8 to 1.9 m/s (according to Equation (33)) for frequencies between 100 and 1000 Hz (see Figure 7), which is in agreement with the reviewed studies [27,47,48,52,59,60,61].

The shear modulus contrast ratio between cancerous and normal tissue was chosen to be 1.2, based on the minimum ratio found by Woo et al. [48] for a Gleason score 6 tumour. The value of α can be considered nearly the same for normal and cancerous tissue, according to the results from the dynamic mechanical analysis by Zhang et al. [59]. For both μ and η, the ratio between cancerous and normal tissue was assumed to be the same. The values used for the shear KVFD parameters are summarised in Table 2.

### 3.3. Numerical Dispersion and Stability Analysis

Numerical modelling inevitably adds numerical dispersion to the natural dispersion of the viscoelastic medium. In order to study this phenomenon, a continuous monochromatic plane shear wave propagation was simulated. The velocity measured from the simulation was compared against the theoretical velocity derived from Equation (33). The number of discretisation elements per wavelength is key for controlling the numerical dispersion. Accordingly, normal prostatic tissue was used as the medium of propagation, since it experiences shorter wavelengths compared to cancerous tissue. The frequency of the excitation varied from 100 to 1000 Hz. Δr and Δz were given the same values, from 70 μm to 6000 μm. Δt was set as 20 μs after an initial estimate. The effective memory length *L* was set at its maximum value corresponding with the total time of propagation, 20 ms.

**Figure 8:**
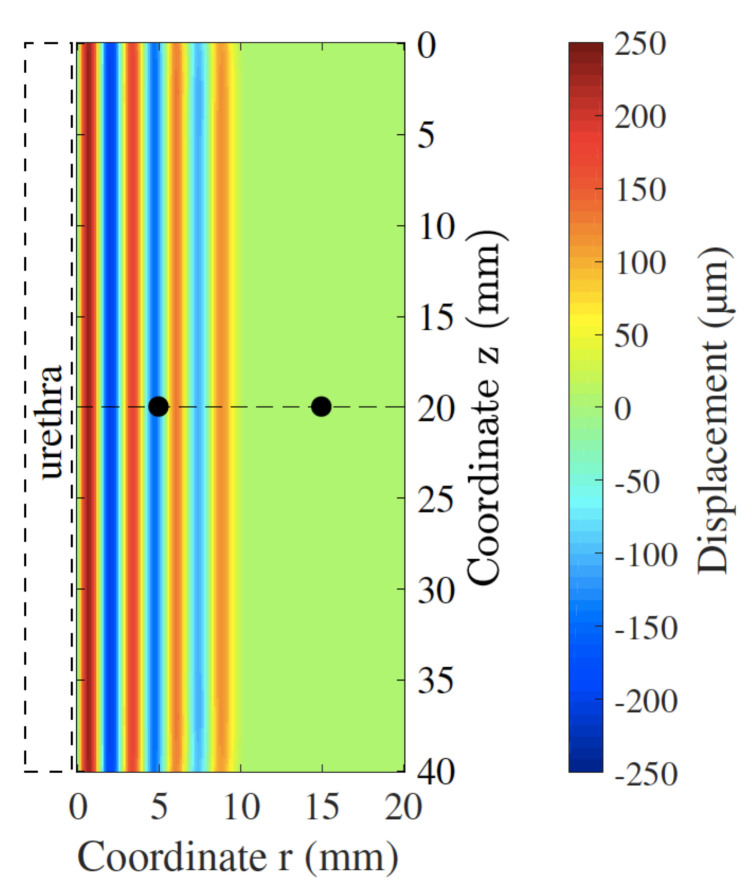
Displacement field produced by a monochromatic shear wave of 700 Hz at 5 ms after the start of the simulation. The black dots show the points where the measurements for calculating the shear velocity were taken from.

2D FDTD simulations were carried out under the described setup. No instabilities were observed during the simulation. Shear velocity was calculated using a time-to-peak approach at two points located in the same radial coordinate (located in dashed black line in Figure 8 at *z* = 20 mm). The two points were located at 5 and 15 mm respectively from the urethral wall.

**Figure 9:**
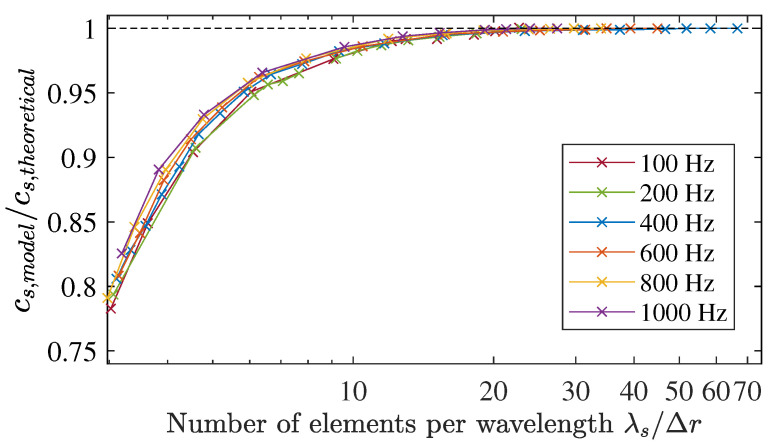
Numerical shear wave velocity dispersion as a function of the number of elements per wavelength (λ/Δr). Cross marks represent the normalised velocity by the theoretical velocity (Equation (33)).

The calculated shear velocity from the time-to-peak measurements in the 2D model was normalised by the theoretical shear KVFD velocity (Equation (33)). These normalised values were expressed as a function of the number of Δr elements per wavelength (λ/Δr) at six frequencies: 100, 200, 400, 600, 800, and 1000 Hz. Results are shown in Figure 9, where the numerical dispersion effect is clearly observable. Numerical errors decrease when increasing the number of elements per wavelength, with an identical tendency at all frequencies tested. Values ranging from 18 elements per wavelength showed acceptable low levels of errors. Specifically, 18 and 25 elements per wavelength yielded errors below 0.42% and 0.25% respectively.

As mentioned in Section 2.2.2, the smaller the value of Δt, the more accurate the finite-difference approximation of the time derivatives. However, Δt has a huge impact on the computational overhead of the simulations. For this reason, a trade-off between accuracy and computational cost was sought. Several values of Δt were tested considering the most computationally demanding scenario, i.e., the propagation in cancerous tissue and at the highest frequency (in this case 1000 Hz). Both Δr and Δz were set to provide a minimum of 18 elements per wavelength. By a trial-and-error process, the stability of the model was ensured by using Δt values lower than 25 μs.

### 3.4. Analysis of the Short-Memory Principle

In order to obtain an optimum *L*, a convergence study was carried out. The short-memory parameter *L* varied from 0 to *N*, the total number of time discretisation elements. *L* = 0 means that none of the previous states is used, whereas *L* = *N*, the reference scenario, means that all the previous states are considered. The Euclidean distance (also known as $l^{2}$-norm) was used to measure the difference between each simulation and the reference scenario.

Four simulations of plane monochromatic shear waves were performed for each of the two types of tissue (normal and cancerous) using frequencies from 100 to 1000 Hz and a total time of simulation of 20 ms. Measurements of the displacement generated were taken at 15 mm from the urethral wall on a centred line. Δr and Δz were set to provide a minimum of 18 elements per wavelength. Δt was set as 20 μs.

**Figure 10:**
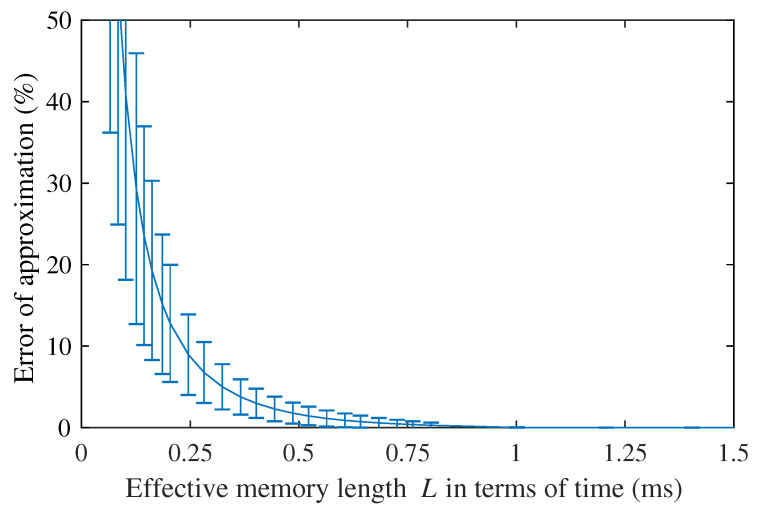
Results from the convergence study for optimising *L*, expressed as the error of approximation due to *L* relative to simulations with L=N. Data are shown in terms of mean and standard deviation values.

Results are shown in Figure 10. Average results were expressed as the error of the approximation relative to the reference case L=N. The *L* parameter was taken in terms of time. The error of approximation converged to 0% at around 0.9 ms.

### 3.5. Simulation of an Extreme Clinical Scenario

The description and values of the remaining model parameters for the particular clinical scenario are listed in Table 3. A general probe setup was chosen, comprising one emitter located at the upper *z* position in the urethra and 32 receivers uniformly distributed along the remaining urethral wall. The excitation signal was set as a Gaussian monocycle with a centre frequency of 700 Hz and maximum amplitude of 0.3 radians, which is equivalent to 1 mm of displacement at the emitter surface of contact with the urethral wall. Δr and Δz were both set at 150 μm. The value of 20 μs chosen for Δt ensured numerical stability. And $t_{L}$, the time associated to *L*, was set at 1 ms.

**Figure 11:**
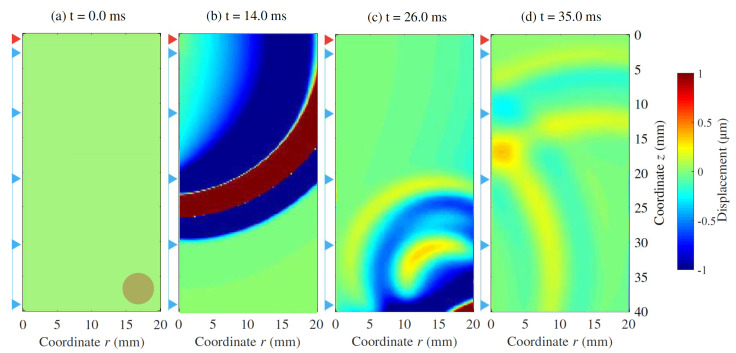
Displacement field during the wave propagation in the clinical scenario, at several time instants. Emitter as a red triangle. Array of 32 receivers uniformly distributed along the urethral wall as blue triangles. Rounded tumour of 4.0 mm as a dark shaded circle. Readout at each receiver’s location is shown in Figure 6.

Figure 11 shows four instants of the wave propagation. The expected propagation of the wave was clearly observable. Refraction and reflection phenomena were also noticeable after the wavefront reached the stiff area.

**Figure 12:**
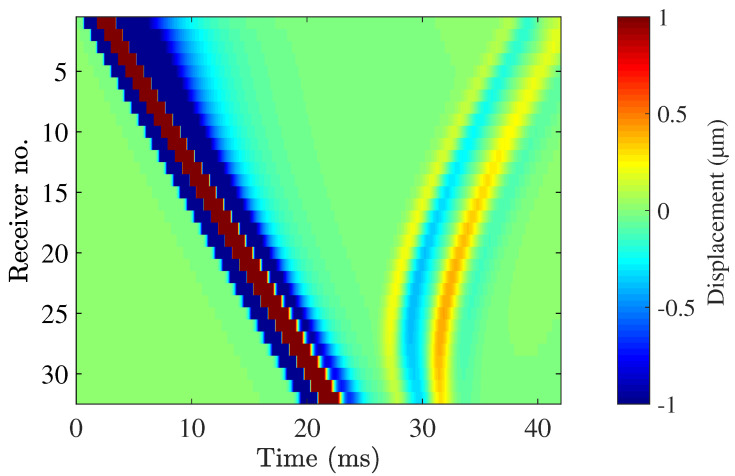
Displacement measured at each receiver’s location over the total time of simulation of the clinical scenario analysed (shown in Figure 6). The position of each receiver is shown in the vertical axis.

The displacement recorded at each receiver’s location is shown in Figure 12. The direct wave propagation along the surface of the urethral wall was detected at all the receivers. The direct propagation is observable in Figure 12 between 0 and 24 ms. The reflection of the shear wave is observable between times 26 and 42 ms. The peak-to-peak displacement of the reflected wave was in the order of 2 μm.

## 4. Discussion

A wave propagation model in fractional viscoelastic media for a new transluminal procedure has been presented in this article. The model implements a KVFD constitutive law, which allows a continuous range of exponents for modelling power law absorption. To the best of the authors’ knowledge, this article shows the first application of a KVFD law for numerically modelling shear wave propagation in a transluminal configuration. The model will be a fundamental part in the future development of any image reconstruction strategy for the new transluminal procedure.

The model was not validated against other computational studies due to the lack of compatible work. One study that modelled the propagation of shear waves in 1D implementing a KVFD constitutive law was found [27]. However, the shear waves from that study were generated by ARF, which implies that the direction of particle vibration was not compatible with that generated by the proposed transluminal approach. A second and more recent study [62] used a 1D semi-analytical model for ARF-generated shear wave propagation using KVFD data extracted from the mechanical characterisation of viscoelastic phantoms. However, apart from occurring in the same incompatible direction of particle vibration, the model assumed negligible wave velocity dispersion, which might be acceptable for a short propagation length but not for the transluminal procedure here presented. As an alternative method of model validation, an experimental strategy using high-speed camera testing will be undertaken in a follow-up study.

Scattering from a prostate tumour was used for both testing the wave propagation model and illustrating an example application of potential interest. The clinical scenario comprised a tumour with the minimum characteristics to be considered of clinical relevance, to be specific, a tumour of 4 mm in diameter and Gleason score 6. According to the literature, this type of tumour shows the lowest change in elasticity compared with normal tissue [47,48,49], therefore generating the weakest level of scattered energy among all the clinically relevant type of tumours. Furthermore, it is obvious that the distance between the emitter source and the tumour is one of the factors that compromises the sensitivity of the technique due to attenuation. To consider this limiting factor, the tumour was placed at the longest expected distance from the emitter’s location. The emission was assumed viable as achieved in other non-related experimental studies using rotational oscillatory disks as emitters [31,32].

The results show that shear waves were satisfactorily transmitted through the urethral wall. The scattered energy from the tumour reached the urethral wall, inducing particle displacement in the order of a micrometer (see Figure 6), which can be considered detectable by current elastography techniques [27,63,64]. Yet, the results must be taken prudently as the prostate model was built with limited data in terms of geometry and real values for the KVFD parameters. Furthermore, a realistic outcome will also depend on the performance of the receivers, the real structural noise of the prostate, and the noise introduced by the transluminal device itself. In any case, larger amplitude echoes can be expected from higher clinically relevant tumours, i.e., those showing a larger size and higher Gleason score. Apart from a stiff tumour, no other type of elasticity heterogeneity was considered in the simulation. The presence of other pathologies and their effect on the wave propagation will need to be addressed in future investigation. In summary, prostate cancer detection seems to be a potential candidate application for the proposed transluminal elastography approach, as the level of achieved sensitivity is enough to pick up changes in the tissue elasticity due to the presence of a tumour.

The transluminal approach may provide some interesting advantages when compared with current extra-corporeal elastography methods, for instance, reaching deep body structures while avoiding obstacles such as bones or other cavities. Unlike most of the current elastography techniques, the transluminal approach does not use ARF to generate the shear waves, which ensures a lower thermal index. In the example shown, the maximum amplitude of particle displacement was 1 mm, at the contact surface between emitter and urethral wall. The maximum shear strain generated was 4.5%, which is within the linear elastic regime for most soft tissues, this is 5–10%, and is below the irreversible damage threshold [65]. The amplitude of the wave rapidly decreased due to attenuation. The maximum amplitude of the reflected wave was in the order of a few micrometers, which is in agreement with other elastography applications that are in clinical use. An additional specific advantage of the transurethral application is that the rectal passage remains free, so transrectal therapies, such as HIFU ablation of prostate cancer, can be simultaneously carried out.

The frequency of the shear wave generated in the transluminal approach is determined by the frequency of the driving signal of the emitter. In the case of most of the commercially available SWE systems, the peak frequency of the shear waves is not higher than 400–500 Hz, in part, due to the limited power output of standard ultrasound equipment being used [33]. With the transluminal approach, shear waves at a higher frequency may be generated as long as the rotational actuator provides high torque and the mechanical contact between disk emitter and lumen wall is perfect. Despite this, the functional frequency range will have to be experimentally investigated for each particular application. Additionally, current elastography methods rely on multiple 2D scans to image volumes [19,50]. In the case of the proposed transluminal approach, a simultaneous 3D scan modality would be theoretically possible as the source will produce shear waves pseudo-spherically and a cylindrical arrangement of receivers will sense waves coming from the entire volume.

The proposed transluminal method is not exempt of possible limitations. However, at this preliminary stage, only limitations associated with the wave generation, propagation, and interaction with the medium can be determined. As discussed before, one limiting factor is the distance between the emitter and region of altered elasticity. A larger distance of propagation implies greater attenuation, which could lead to a poor SNR. In the opposite scenario, where the region of altered elasticity is close to the emitter, the signals due to the direct propagation along the lumen wall overlap with the echoes coming from the region of altered elasticity. This will introduce further complexity for the inverse approach for reconstructing the location and mechanical properties of the region of altered elasticity. Strategies to overcome these two challenges can be based on the use of multiple emitters. Another additional limiting factor, also mentioned before, is the quality of the mechanical contact between the emitter and lumen wall. In the simulated scenario, a perfect contact was assumed, which in reality can be challenging to achieve due to the irregularities of the lumen geometry, the presence of fluid on the contact surface, and the fact that the diameter of the transluminal probe has to be smaller than the diameter of the lumen to allow the insertion. Despite this, case dependent strategies to maximise the quality of the mechanical contact can be investigated. In the prostate case, suction can be applied so the prostatic urethra collapses onto the transluminal device [58]. For intravascular imaging, a compliant stent-like emitter can be used to transmit the oscillatory rotation after increasing in diameter and making contact with the vessel wall. Research on the contact mechanics and the use of multiple emitters will be part of future investigations.

The technical aspect of the transluminal procedure to reach clinical practice are also specific for each application. In the case of prostate cancer detection, the transluminal probe will form part of a urethral Foley-type catheter. The catheter will be lubricated to reduce patient discomfort during insertion. After anchoring an inflatable balloon located at the tip of the catheter to the bladder neck, a sheath will be retrieved to expose the probe segment of the catheter. Suction will be applied to aspire the fluid in the prostatic urethra and to ensure a mechanical lock between the probe and urethral wall. Furthermore, if the cost of the sensing components are kept low, the whole catheter-probe can be disposable, and sterilisation will not be required. This will facilitate the use of the transurethral procedure in standard clinical facilities, as an advantage against transrectal elastography which is performed in surgery rooms and requires full sedation of the patient.

## 5. Conclusions

This article showed the application of a KVFD-based wave propagation model to a new transluminal procedure for performing elastography. The model is planned to be part of an image reconstruction algorithm in future work. The model uses a FDTD scheme for solving the KVFD-based wave equation. The KVFD model belongs to the family of fractional lineal viscoelastic rheological models. In the last two decades, the use of fractional rheological models for modelling elastography have gained relevance. These models could accurately reproduce in the time and frequency domains the cumulative effect of multiple relaxation processes found in soft tissue mechanics. For wave modelling, this implies that fractional viscoelastic models could model power law absorption with variable exponent, something that could not be achieved by using classical viscoelastic models. The developed wave propagation model provided a useful tool to establish the feasibility of transluminal procedures based on wave propagation and interaction with areas of altered viscoelastic property outside the lumen.

The new transluminal procedure opens a new way of performing elastography from body lumina. Prostate cancer detection using the transluminal method through the urethra stands as a first potential application. Further exploration to analyse other potential applications is encouraged, as other medical applications, such as the characterisation of atherosclerotic plaques, might also be benefited from the proposed transluminal approach.

## 6. Patents

Patent no. ES2687485A1—PCT/ES2018/070243 [29] is result from the work reported in this manuscript.

## Figures and Tables

**Table 1:** Values of the spatial dimensions of the model domain for the particular clinical scenario.

Parameter	Description	Value
$r_{d}$	Radial dimension of the domain	20.00 mm
$z_{d}$	Depth dimension of the domain	40.00 mm
$r_{u}$	Radius of the urethra	3.25 mm

**Table 2:** Values for the three KVFD shear parameters proposed for modelling all tissue conditions in the Finite Difference Time Domain (FDTD) wave propagation model.

	Type of Prostatic Tissue	
KVFD Parameter	Normal	Cancerous
μ (kPa)	3.0	3.6
η (Pa·s${}^{\alpha }$)	35.0	42.0
α	0.35	0.35

**Table 3:** Values of the model parameters for the particular clinical scenario. PML: Perfectly Matched Layer.

Param.	Description	Value
	*Discretisation parameters*	
Δr	*r* spatial dimension interval	150.00 μm
Δz	*z* spatial dimension interval	150.00 μm
Δt	time interval	20.00 μs
$t_{T}$	Total time of simulation	42.00 ms
$t_{L}$	Time reference for *L* param.	1.00 ms
$n_{PML}$	Number of PML elements	60
	*Probe setup*	
$n_{e}$	Number of emitters	1
$z_{e}$	*z* coordinate of the emitter	1.00 mm
$l_{e}$	Length of the emitter	2.00 mm
$f_{e}$	Centre frequency of the excitation	700 Hz
$a_{e}$	Max. amplitude of the excitation	0.30 rad
$n_{r}$	Number of receivers	32
$\Delta z_{r}$	Distance between receivers	0.80 mm
$l_{r}$	Length of each receiver	0.50 mm
	*Tumour features*	
$r_{c}$	*r* coordinate of the tumour centre	16.00 mm
$z_{c}$	*z* coordinate of the tumour centre	36.00 mm
${}_{c}$	Diameter of the tumour	4.00 mm

## Abbreviations

The following abbreviations are used in this manuscript:

KVFD	Kelvin Voigt Fractional Derivative
FDTD	Finite Difference Time Domain
SE	Strain Elastography
ARF	Acoustic Radiation Force
SWE	Shear Wave Elastography
KV	Kelvin Voigt
SNR	Signal-to-Noise Ratio
SDUV	Shear Wave Dispersion Ultrasound Vibrometry
PML	Perfectly Matched Layer
GL	Grünwald-Letnikov
BPH	Benign Prostatic Hyperplasia
HIFU	High Intensity Focused Ultrasound

## Appendix A. FDTD Discrete Equations

This appendix describes the derivation of the discrete equations used in the wave propagation model. The discrete equations are derived after applying the space-time discretisation to the domain, and the FDTD expressions to the equations that describe the physical phenomenon of wave propagation in a KVFD viscoelastic medium.

First, the equations of the problem are split and the PML parameters are incorporated following the procedure for cylindrical coordinates developed by [43]. The split expressions are adapted to a KVFD constitutive law.

The conservation of momentum equation in cylindrical coordinates (Equation (22)) is split in the following expressions:

(A1) $$a_{r}\frac{\partial^{2}u_{\theta (r)}}{\partial t^{2}}+\omega_{r}\frac{\partial u_{\theta (r)}}{\partial t}=\frac{\partial \sigma_{r\theta }}{\rho \partial r}$$

(A2) $$A_{r}\frac{\partial^{2}u_{\theta (\theta )}}{\partial t^{2}}+\Omega_{r}\frac{\partial u_{\theta (\theta )}}{\partial t}=2\sigma_{r\theta }$$

(A3) $$a_{z}\frac{\partial^{2}u_{\theta (z)}}{\partial t^{2}}+\omega_{z}\frac{\partial u_{\theta (z)}}{\partial t}=\frac{\partial \sigma_{\theta z}}{\rho \partial z}$$

where $u_{\theta }$ = $u_{\theta (r)}+u_{\theta (\theta )}+u_{\theta (z)}$ according to the notation employed by [43]. $a_{r}$, $A_{r}$, $a_{z}$, $\omega_{r}$, $\Omega_{r}$, and $\omega_{z}$ are the PML variables as described by [43]. $a_{r}$ = $a_{z}$ = 1, whilst $A_{r}$ = 1/r. $\omega_{r}$, $\Omega_{r}$ and $\omega_{z}$ are the absorbing parameters. $\Omega_{r}$ = 0, whilst $\omega_{r}$ and $\omega_{z}$ are 0 in the space domain of the problem and a parabolic law that shows values from 0 to 1.6ω, with ω the centre frequency of the excitation. The value of 1.6 was obtained by a trial-and-error process.

The strain-displacement relationship (Equations (23) and (24)) are fused into the KVFD constitutive law equations (Equations (19) and (21)), thus reducing the memory required for computing the algorithm. Then, the resulting equations are differentiated respecting time and then split in the following expressions:

(A4) $$a_{r}\frac{\partial \sigma_{r\theta (r)}}{\partial t}+\omega_{r}\sigma_{r\theta (r)}=\mu \frac{\partial }{\partial t}\left(\frac{\partial u_{\theta }}{\partial r}\right)+\eta \frac{\partial^{\alpha +1}}{\partial t^{\alpha +1}}\left(\frac{\partial u_{\theta }}{\partial r}\right)$$

(A5) $$A_{r}\frac{\partial \sigma_{r\theta (\theta )}}{\partial t}+\Omega_{r}\sigma_{r\theta (\theta )}=-\mu \frac{\partial u_{\theta }}{\partial t}-\eta \frac{\partial^{\alpha +1}u_{\theta }}{\partial t^{\alpha +1}}$$

(A6) $$a_{z}\frac{\partial \sigma_{\theta z(z)}}{\partial t}+\omega_{z}\sigma_{\theta z(z)}=\mu \frac{\partial }{\partial t}\left(\frac{\partial u_{\theta }}{\partial z}\right)+\eta \frac{\partial^{\alpha +1}}{\partial t^{\alpha +1}}\left(\frac{\partial u_{\theta }}{\partial z}\right).$$

Then, the FDTD expressions are applied to approximate the above shown equations. Equation (28) for the spatial derivatives, Equation (29) for the first temporal derivatives, Equation (30) for the second temporal derivatives, and Equation (31) for the fractional temporal derivatives. The derived expressions listed below are the discrete equations that are implemented in the algorithm.

Equation (A1) yields the following discrete equation:

(A7) $$\begin{array}{cc} {\left.u_{\theta (r)}\right|}_{i,j}^{n} & =H_{u(r)}{\left.u_{\theta (r)}\right|}_{i,j}^{n-1}-M_{u(r)}{\left.u_{\theta (r)}\right|}_{i,j}^{n-2}\\ \; & +M_{u(r)}\frac{\Delta t^{2}}{\rho a_{r}\Delta r}\left({\left.\sigma_{r\theta }\right|}_{i+\frac{1}{2},j}^{n-1}-{\left.\sigma_{r\theta }\right|}_{i-\frac{1}{2},j}^{n-1}\right) \end{array}$$

with:

(A8) $$M_{u(r)}=\frac{1}{1+\frac{w_{r}\Delta t}{a_{r}}}$$

(A9) $$H_{u(r)}=\left(2+\frac{w_{r}\Delta t}{a_{r}}\right)M_{u(r)}.$$

The notation ${\left.g\right|}_{i,j}^{n}$ indicates that the *g* function is evaluated at the spatial (i,j) grid node for the *n*th time step.

Equation (A2) yields the following discrete equation:

(A10) $$\begin{array}{cc} {\left.u_{\theta (\theta )}\right|}_{i,j}^{n} & =H_{u(\theta )}{\left.u_{\theta (\theta )}\right|}_{i,j}^{n-1}-M_{u(\theta )}{\left.u_{\theta (\theta )}\right|}_{i,j}^{n-2}\\ \; & +M_{u(\theta )}\frac{\Delta t^{2}}{\rho A_{r}}\left({\left.\sigma_{r\theta }\right|}_{i+\frac{1}{2},j}^{n-1}+{\left.\sigma_{r\theta }\right|}_{i-\frac{1}{2},j}^{n-1}\right) \end{array}$$

with:

(A11) $$M_{u(\theta )}=\frac{1}{1+\frac{W_{r}\Delta t}{A_{r}}}$$

(A12) $$H_{u(\theta )}=\left(2+\frac{W_{r}\Delta t}{A_{r}}\right)M_{u(\theta )}.$$

Equation (A3) yields the following discrete equation:

(A13) $$\begin{array}{cc} {\left.u_{\theta (z)}\right|}_{i,j}^{n} & =H_{u(z)}{\left.u_{\theta (z)}\right|}_{i,j}^{n-1}-M_{u(z)}{\left.u_{\theta (z)}\right|}_{i,j}^{n-2}\\ \; & +M_{u(z)}\frac{\Delta t^{2}}{\rho a_{z}\Delta z}\left({\left.\sigma_{\theta z}\right|}_{i,j+\frac{1}{2}}^{n-1}-{\left.\sigma_{\theta z}\right|}_{i,j-\frac{1}{2}}^{n-1}\right) \end{array}$$

with:

(A14) $$M_{u(z)}=\frac{1}{1+\frac{w_{z}\Delta t}{a_{z}}}$$

(A15) $$H_{u(z)}=\left(2+\frac{w_{z}\Delta t}{a_{z}}\right)M_{u(z)}.$$

Equation (A4) yields the following discrete equation:

(A16) σrθ(r)i+12,jn=Hσ(r)σrθ(r)i+12,jn−1+Mσ(r)μarΔruθi+1,jn−uθi,jn−Mσ(r)μarΔruθi+1,jn−1−uθi,jn−1+Mσ(r)ηarΔrΔtα∑k=0L(−1)kα+1kuθi+1,jn−k−uθi,jn−k

with:

(A17) $$M_{\sigma (r)}=\frac{1}{1+\frac{w_{r}\Delta t}{2a_{r}}}$$

(A18) $$H_{\sigma (r)}=\left(1-\frac{w_{r}\Delta t}{2a_{r}}\right)M_{\sigma (r)}.$$

Equation (A5) yields the following discrete equation:

(A19) σrθ(θ)i+12,jn=Hσ(θ)σrθ(θ)i+12,jn−1−Mσ(θ)μ2Aruθi+1,jn+uθi,jn−Mσ(θ)μ2Aruθi+1,jn−1+uθi,jn−1−Mσ(θ)η2ArΔtα∑k=0L(−1)kα+1kuθi+1,jn−k+uθi,jn−k

with:

(A20) $$M_{\sigma (\theta )}=\frac{1}{1+\frac{W_{r}\Delta t}{2A_{r}}}$$

(A21) $$H_{\sigma (\theta )}=\left(1-\frac{W_{r}\Delta t}{2A_{r}}\right)M_{\sigma (\theta )}.$$

Equation (A6) yields the following discrete equation:

(A22) σθz(z)i,j+12n=Hσ(z)σθz(z)i,j+12n−1+Mσ(z)μazΔzuθi,j+1n−uθi,jn−Mσ(z)μazΔzuθi,j+1n−1−uθi,jn−1+Mσ(z)ηazΔzΔtα∑k=0L(−1)kα+1kuθi,j+1n−k−uθi,jn−k

with:

(A23) $$M_{\sigma (z)}=\frac{1}{1+\frac{w_{z}\Delta t}{2a_{z}}}$$

(A24) $$H_{\sigma (z)}=\left(1-\frac{w_{z}\Delta t}{2a_{z}}\right)M_{\sigma (z)}.$$
